# Is telepsychiatry care a realistic option for community mental health services during the COVID-19 pandemic?

**DOI:** 10.1177/0004867420937788

**Published:** 2020-06-22

**Authors:** Zlatan Zulfic, Dennis Liu, Cynthia Lloyd, Jacqueline Rowan, Klaus Oliver Schubert

**Affiliations:** 1 Northern Community Mental Health Service, Northern Adelaide Local Health Network, Salisbury, SA, Australia; 2 Discipline of Psychiatry, Adelaide Medical School, The University of Adelaide, Adelaide, SA, Australia

To the Editor

People with severe mental illnesses have higher rates of smoking and medical comorbidities which are known to increase risk of serious COVID-19 illness including metabolic syndrome, chronic lung disease and cardiac disease (Firth et al., 2019). The current COVID-19 pandemic has necessitated a significant review of practices for community mental health teams in order to manage the risk of infection to patients and staff.

Our team conducted an audit of 314 community patients to examine the potential implications of a move to predominantly telephone support. We identified 118 patients (38%) as having significant medical comorbidities or advanced age (>55 years), placing them at higher risk of adverse outcomes in case of COVID-19 illness. In all, 21 (7%) did not have access to a phone, and a further 58 (18%) were deemed unreliable in responding to contact over the phone based on past clinician experience. The majority of these difficult-to-reach patients had a diagnosis of schizophrenia or schizoaffective disorder (83%), and 75% were considered high risk for COVID-19 complications. In addition to those who lack access to the technology required, there was a significant group of patients that require regular face-to-face reviews, including the 91 patients (29%) who are treated with depot medications and 71 (23%) taking clozapine.

Prior to and during the current pandemic, our service has offered mobile phones to 17 of our current patients. Twelve (71%) declined the offer or gave the phone away. A recent study of patients in our region showed limited access and confidence in using technology among people with schizophrenia, which could be a barrier to online interventions (Wong et al., 2020). There is also the potential for additional clinical risks when using telepsychiatry in an already high-risk population, especially for those presenting in crisis (Cowan et al., 2019).

Although the move by governments and health services to encourage increased telehealth take-up during this period is important, our data suggest that there are significant barriers for our core cohort of patients. In response to the current situation, service delivery should be carefully planned to emphasise patient education, social supports and maintaining therapeutic adherence. Clinician safety and well-being should be paramount when face-to-face contact is required. A dynamic and effective mental health system response is essential not only for the health and well-being of people with severe mental illness but also for mitigating the spread of the infection in the community.

## References

[bibr1-0004867420937788] Cowan KE McKean AJ Gentry MT, et al (2019) Barriers to use of telepsychiatry: Clinicians as gatekeepers. Mayo Clinic Proceedings 94: 2510–2523. 31806104 10.1016/j.mayocp.2019.04.018

[bibr2-0004867420937788] Firth J Siddiqi N Koyanagi A, et al (2019) The Lancet Psychiatry Commission: A blueprint for protecting physical health in people with mental illness. Lancet Psychiatry 6: 675–721. 31324560 10.1016/S2215-0366(19)30132-4

[bibr3-0004867420937788] Wong KTG Liu D Balzan R, et al (2020) Smartphone and internet access and utilization by people with schizophrenia in South Australia: Quantitative survey study. JMIR Mental Health 7: e11551. 32012068 10.2196/11551 PMC7013647

